# Proceedings of the Pathological Society of Philadelphia

**Published:** 1859-01

**Authors:** 


### Art. V.—*Proceedings of the Pathological Society of Philadelphia.*

Wednesday Evening, October 13th, 1858.

The President, Dr. Gross, in the chair.

*Tubercles in the Cerebrum and Cerebellum.*—Dr. Packard, in presenting these specimens, said:—For notes of this case, I am indebted to Dr. F. W. Lewis and Dr. Dunton, who attended the patient.

James Gray, aged six, of a hereditary scrofulous habit, slipped, or was pushed, from a sidewalk, in September, 1857, and in his fall struck the back of his head forcibly against the curb-stone. The blow stunned him for a time, and for several weeks he had occasionally severe attacks of pain in the occiput. He however continued otherwise well until the latter part of December, when the paroxysms of pain became more frequent and intense, recurring several times daily.

From this time he grew worse and worse until April 26th, 1858, when he was admitted into the Children's Hospital, under the care of Dr. Penrose. On admission his symptoms were as follows:—Head rather large, body and limbs decidedly emaciated; expression of countenance "cerebral," with a marked amaurotic stare, and considerable dilatation of both pupils, particularly of the *left;* complexion somewhat waxy, with irregular flushing of one or both cheeks; decubitus dorsal, with slight flexion backward of the head. His intelligence was not impaired, nor were his special senses, except, perhaps, that of vision. He complained at intervals of intense pain in the occiput, and during these paroxysms he would utter short, sharp cries; their duration was usually two or three minutes. They occurred several times in the twenty-four hours, and were most aggravated at night. The temperature of his skin was natural; his head was not abnormally heated; his respiration was unaffected; his pulse regular, full, about 80 per minute. His appetite was voracious, but his digestion rather sluggish, and his bowels constipated.

He was removed from the hospital in about ten days, having derived no benefit during his sojourn.

From this date the unfavorable symptoms made very rapid progress. His emaciation became very considerable, especially in the body and extremities; his complexion more waxy, and the irregular flushing of one or both cheeks more vivid. In addition to the decubitus before mentioned, his knees became drawn up. His intellect was still clear; the amaurosis was now complete. There was no unusual heat of head, but the temporal and carotid arteries throbbed very forcibly during the paroxysms of pain, which amounted to agony. These attacks now recurred about every half hour, with a strongly-marked tendency to remission on the alternate days.

His digestion, respiration, and pulse, (except during the attacks,) continued much as before; his appetite was capricious, although mostly very voracious. It was always observable that the act of mastication continued for some time after his food had been swallowed. Owing to his inordinate appetite he had frequent dysenteric attacks; toward the close he began to pass his urine and stools involuntarily. The urinary secretion was generally copious, high-colored, of neutral reaction, and showed a tendency to deposit phosphates shortly after being passed. No priapism was at any time observed.

About the beginning of July, the emaciation and debility became so extreme that his death was daily looked for. He rarely moved, but lay with his knees drawn up, his head thrown back, his eyes open, his pupils (especially the *left*) enormously dilated, the conjunctivæ inflamed and covered with a viscous secretion. His pulse became more frequent and irregular, and he could hardly be seen to breathe at all. From this apparently almost lifeless condition he would at times rouse himself and call for food.

July 23d.—He cries at intervals; answers questions, but never talks. Flexors of both upper and lower extremities in a state of tonic contraction. He has some difficulty in swallowing; tongue red and covered with *muguet.* His bowels are quiet, and have been for three days past; previous to this he had diarrhœa.

July 25th.—He has less difficulty in swallowing; vomits occasionally; was several times convulsed during the night.

July 30th.—An ulcer appeared on the right cornea. He gapes continually.

August 17th.—Emaciation much increased; contraction of flexors more decided.

August 26th.—Diarrhœa; abdomen much retracted, with enlargement of its superficial veins.

August 30th.—Has had no convulsions for the last two weeks, but lies moaning; occasionally his breathing seems to stop entirely. His death occurred on the 3d of September.

By the kindness of Dr. Dunton, I assisted at the autopsy, which was made thirty-three hours after death; the head only was examined.

Brain softened, and a very large quantity of serous effusion in the ventricles. The cineritious layer was very thick. In each lobe of the cerebellum was found a tumor of about the size of a walnut, rounded, deep-yellow, of firm consistence, and presenting traces of a concentric formation. A similar tumor, but of smaller size, was taken from the posterior portion of the right cerebral hemisphere.

Under the microscope, the structure of these tumors appeared to be tuberculous. Some of the elements observed bore some resemblance to those seen in amyloid formations in the brain.

A case remarkably similar, in some respects, to the one I have now reported, was published by Dr. Pepper, in the *Medical Examiner* for December, 1856. In that case there was, however, no injury known to have been received, and the course of the disease was much more rapid, (only four months;) the attacks were also much more epileptiform in their character. In my notes taken at the time of the autopsy, some particulars are set down, which were omitted by Dr. Pepper because they had no direct bearing upon the point discussed in his article.

The calvarium being removed, the skull was found to be excessively thin—having not more than half its proper thickness. The inner surface was rough and vascular, and much darker than normal; on the outside, a patch, reaching from the coronal suture back as far as the division of the skull, (in removing the skull-cap,) and on each side to the line of attachment of the temporal fascia, was discolored in much the same way, and the vessels most beautifully injected.

On removing the brain with the dura mater, the base of the skull had a similar appearance, being over the sphenoidal cells so thin as to feel like a mere membrane. This change was especially well marked in the channels for the lateral sinuses, which seemed deepened as if by an erosive action. The surface of the brain was much congested, and a large quantity of clear serum escaped when the dura mater was cut into.

The cerebellum was softened down, and within its substance, just between the two lobes, was a tuberculous deposit of the shape of an English walnut; this mass weighed half an ounce, and measured an inch and a quarter in diameter.

*Pneumonia and Pleuritic Effusion occurring in Enteric Fever, with signs of Perforation of the Intestine, but without any post-mortem evidence of this lesion.*—DR. HALL exhibited, for Dr. Albert H. Smith, an ulcerated intestine and inflamed lung, removed from the body of Chas. Huttner, aged thirty-two, German, who was admitted on the 11th of September into the Pennsylvania Hospital. He had been taken sick, about two weeks previously, with enteric fever, and passed through the ordinary course of the disease without having, as far as could be learned, any remarkable or unusual symptoms. On the 27th of September he was convalescent; the rose-spots had disappeared; the pulse was regular; the bowels very slightly inclined to diarrhœa; no tenderness of abdomen; no chest symptoms, except an occasional appearance of sonorous rhonchi; and although quite weak, his strength was gradually returning and all the functions being well performed.

In this way he continued (great care having been taken to guard him from exposure to cold, and from the use of any solid or irritating diet) until the morning of the 6th inst., when he was found with a cold, clammy skin, the pulse 160, feeble and irritated, the countenance anxious and distressed, with great pain in abdomen, slight tympanites, and a tendency to delirium. On the supposition that perforation had taken place, he was placed upon beef-essence and milk-punch; dry cups were applied to the abdomen, and sufficient laudanum was given to narcotize. The symptoms continued for twenty-four hours, when they entirely subsided. The skin became warm and dry, the pulse fell to 110, the pain in abdomen ceased, no tympanites was present, and the patient felt altogether more comfortable, although greatly prostrated. The stimulants and nourishment were continued.

In about twelve hours another set of symptoms had developed themselves. In addition to the dry, hot skin and frequent pulse above mentioned, there came on, for the first time, violent chest symptoms: great oppression and dyspnœa, constant cough, with expectoration of bloody sputa; entire flatness of the posterior portion of both lungs and the lower lobes anteriorly; subcrepitant rhonchi on right side, at base; crepitant rhonchi and friction sound on left side. The patient was dry-cupped over the chest, blistered on the left side, and kept constantly turning so as to prevent hypostatic congestion. Mustard poultices were applied to the extremities, and carbonate of ammonia was administered, but he continued to sink from this time, and died on the 9th.

The autopsy showed congestion of the lower part of right lung, with red hepatization of the lower lobe of the left; over the whole pulmonary pleura of the left side there were thick patches of lymph, and in the pleural cavity about a half-pint of serum. There was no other lesion except the ulceration in the small intestines, which was extensive, but appeared to be healing. No evidence of perforation could be detected.

*Phthisis with Paraplegia.*—DR. HALL exhibited, also for Dr. Albert H. Smith, a tuberculous lung and a softened spinal marrow:—M. B., aged fifty-four, was admitted into the Pennsylvania Hospital September 27, 1858, with evident signs of tubercle at the apices of both lungs. He had a cough, and had been gradually losing his strength for six weeks. He was very feeble, almost unable to walk, dragging the right leg in his attempts; the countenance was pale, with a particularly anxious and haggard expression. His symptoms gradually grew worse, until complete paraplegia of the lower limbs ensued. There were no cerebral symptoms; sensation and voluntary motion declined gradually and simultaneously in the limbs; there was no spasmodic action. He died from general prostration, his death being hastened by suffocation from inability to expectorate.

The autopsy showed both lungs infiltrated with tubercles; a cavity existed at the upper portion of the right lung; the heart, brain, and the other viscera were healthy; on removing the arches of the vertebræ, there was found in the arachnoid about six ounces of serous fluid; the pia mater was greatly injected; the congested vessels ran in places into the substance of the cord, which was completely softened.

*Gangrene of the Spleen.*—DR. HALL presented the following paper on gangrene of the spleen, with a water-color drawing of a specimen of the disease:—

Of this abnormal condition of the spleen Rokitansky observes, "that it is as rare an occurrence in the spleen as in the liver; we have had an opportunity of observing it once in a chronic tumor of the spleen, affecting the organ to a considerable extent."

Baillie* remarks "that it is very rare to find the substance of the spleen either in a state of inflammation or suppuration, but such cases have been occasionally observed and related by authors. Instances have also been related where the spleen has been observed to be 'mortified,' but this I should believe to be even much more rare than the former."

As a witness on the other side, Portal† states that "Gangrene of the

* Morbid Anatomy, p. 168. † Anatomie Médicale, tome v. p. 336.

spleen is observed rather frequently, (assez souvent;) it is recognized by the softening of its tissue, and the fetid odor which it exhales; it is the frequent consequence of the inflammation of this viscus, and then the rational symptoms (les symptôms exposés) above mentioned diminish or cease suddenly, and mortal weakness and sinking succeed. It has also been remarked in subjects who have died of malignant fevers."

*Case.*—During my service of House-Surgeon in the Pennsylvania Hospital in 1856, was admitted on the 25th of September a stout, vigorous young German, Michael H——, aged twenty-three, with an extensive gunshot wound of the upper part of the right arm, which had cut clean across the brachial artery at the upper third of the biceps muscle of that side. There was not any bleeding of consequence at the time of admission, although we were told that there had been, an hour before; shortly after the occurrence of the accident, a Spanish windlass had been twisted above the wound, but no ligatures were applied. The brachial artery was tied in the wound, and eight or nine ligatures were put on smaller vessels after admission into the house. The arm from the site of injury down to the fingers was cold; there was no pulsation in the arteries below the wound. A consultation was held, but the man was so depressed from the shock that amputation at that time was out of the question. On the next morning, however, the patient having rallied sufficiently, Dr. Neill (the surgeon on duty) amputated the arm about three inches below the axilla.

October 27th, (a month after the operation.)—Pulse 102; patient feverish, with hot skin and flushed cheeks; great deal of deep suppuration in the stump, an erysipelatous blush on its surface; complains of pain in the right "hypochondrium." [I wish the Members to remark this hypochondriac pain, as I think that it was reflexly sympathetic from the inflamed spleen in the left hypochondrium.]

The next day he had a slight chill, followed by fever and sweating; pulse 99, full and quick; tongue a little furred, yellow; "*pain* still in the right hypochondrium; *increased* by pressure."

November 4th.—My term of service in that ward having expired, I handed over my charge to Dr. George H. Humphreys, my successor in office, still, however, by his kindness, observing with him the progress of the case.

Some time later, unmistakable symptoms of pyaemia developed themselves, became confirmed, and, as usual, in spite of any treatment, carried off the patient in about four weeks' time.

*Sectio cadaveris* ten hours after death.—Rigor mortis well pronounced; body not much emaciated, well formed. Head not permitted to be examined. The bone of the amputated arm presented a ring of necrosis one-quarter of an inch in length. Pus in the veins of the stump. On opening the thorax one quart and a half of straw-colored serum was found in the pleural sac. The lungs were much congested, with localized spots of inflammation ; several small abscesses of one inch in diameter were found in the posterior aspect of the lungs. Liver healthy. The spleen was lobulated ; it measured five and a half inches in length, and four inches in breadth ; form natural, *i. e.* slightly concavo-convex. Color of a bright-red, approaching crimson. On its anterior inferior surface was a patch of gangrene one and a half inches in circumference. This was of a dark, greenish hue ; mottled ; color well defined ; not shading off into the color of the surrounding texture ; flaccid to the touch, with a sense of fluctuation. Odor, garlic-like.

There was a well-marked line of demarcation, of a pinkish color, with numerous vesicles upon its surface. The discoloration was superficial, merely involving the capsule, not going down into the splenic substance ; beneath this was a cavity two inches in depth, filled with a soft chocolate-colored matter. There was a small cherry-like cyst on the omentum, just beneath the spleen. Bladder distended with urine.

As already stated, gangrene of the spleen is of very rare occurrence. Rokitansky* speaks of an inflammation of the spleen, in which "the portion affected is converted into a puriform creamy mass, or into a sanious, greenish, greenish-brown, or chocalate-colored pulp." Magaziner† gives an account of an epidemic gangrene of the spleen that occurred during the year 1831, at Dorfe Mandrowa, Russia, called by him the "Siberian Epidemic," or "Splenitis Gangrenosa."

A male and three females were seized with a burning pain under the præcordial region ; with nausea ; pain in the abdomen, and especially about the spleen. Two died, and a post-mortem examination gave the following appearances : Body of the patient who died on the third day of the disease. "A dark spot on the left side of the abdomen ; traces of inflammation here and there on the peritoneum ; stomach and intestines distended with gas, and inflammation of their external surface ; the spleen, much enlarged and tender ; when divided gave issue to a great deal of dark blood, and presented on its surface *a large* gangrenous point." Nothing abnormal in the head or chest.

The appearances were the same in the body of the other patient.

On the 29th January, 1831, two females were seized with the same symptoms, and the writer now recognized the existence of the "Siberian Epidemic," or "Splenitis Gangrenosa." There is a reference to this Siberian Epidemic in *Canstatt's Jahresbericht*‡ for 1851.

---

* Path. Anat., vol. ii. p. 137, Am. ed.

† Kleinert's Repertoire, from the Russian Military Med. Gazette, No. 3, 1833.

‡ Band vi. p. 15.

Bright* notices the occasional occurrence of gangrene of this organ, and cites the case of Anne Howell, a house-maid, aged twenty-one, received into Guy's Hospital, January 24th, 1829, with symptoms of great depression, in a most reduced state, having been ill some weeks and undergone active treatment for what was supposed to be carditis. On admission there was observed a rapid, weak pulsation of the heart, and frequent vomiting, under which she sunk in about ten days.

*Sectio cadaveris.*—In the abdomen there were some cellular adhesions of long duration between the liver and diaphragm, and between the liver and stomach; on the left side were more recent adhesions in the neighborhood of the spleen, circumscribing a cavity bounded above by the under-surface of the left lobe of the liver, to the inner side by the stomach, and to the outer side by the spleen. This cavity contained an offensive dark-colored matter, with "a strong gangrenous odor," which was produced by the breaking down of a portion of the spleen, extending to no great depth into the organ, the remainder of which was firmer than natural, and of a *red color*.

I wish to call the Society's attention to the red color of the spleen, as it was so well marked in the specimen before them. It was not owing to the action of the air upon the organ, but presented the same hue at the moment of removing the organ from the body. It is undoubtedly the intense red of acute inflammation.

Many cases of disorganized or broken-down spleens are quoted by Lieutaud.† Of course what some of these older writers say on this subject must be received with caution; but still, when they describe the appearances of gangrene, together with "a most offensive odor," it is but right to believe that what they saw was gangrene: "A man‡ died on the eighteenth day of a continued fever; the spleen was tumid, livid, and 'greenish,' from which, when incised, a great quantity of matter like black pitch flowed forth." Also, a case§ in which the spleen was livid and black, a portion cut off, as though amputated. Another,|| in which the spleen was gangrenous and partly destroyed. Finally,¶ a gangrenous and putrid spleen, after a pneumonia.

Cases are cited by Morgagni,** one in which the spleen was impregnated with a gangrenous blackness, and exhaled "a most offensive odor."

The "offensive odor" here mentioned is of more importance as a point of medical evidence than the gangrenous blackness so often dwelt upon.

---

* Guy's Hospital Reports, vol. iii., 1838.

† Historia Anatomico-Medica, tome i. p. 223.

‡ Observat. 958. § Ibid. 961.

|| Ibid. 966. ¶ Ibid. 970.

** Epist. Anat.-Medica, xxv. art. 16.

This black color may exist without gangrene; it should not, taken alone, be considered as a sign of gangrene.* Morgagni† cites a case after peripneumonia:—"The abdomen being opened, a heavy odor was perceptible, as if from inflamed viscera, taking on gangrene. The liver was whitish, but the spleen was affected with a gangrenous blackness."

Dr. Janney, of this city, informs me that he has seen a case in which the spleen was three times its natural size; he cannot recollect how large the gangrenous portion was, as it occurred years ago during an epidemic of intermittent; it had a gangrenous odor, and he had no doubt that it was gangrene.

Briefly to compare other states of the spleen with gangrene, I shall indicate how a lesion which resembles it most—abscess—is found in the organ. Abscesses of the lungs and liver are not unusual after operations, injuries of the head, compound fractures, puerperal phlebitis, and even suppuration around a foreign body; but in the spleen they are not often observed. Speaking of pyæmia, Nélaton‡ remarks the frequency of abscesses of the lungs and liver, but does not notice their presence in the organ under consideration. Mackintosh,§ however, mentions a case of a large abscess of the spleen, after an amputation of the leg. In the fourth volume of the *Transactions of the Pathological Society of London* is reported an abscess of the spleen after puerperal phlebitis.

The splenitis in these cases is consecutive, and owing to an alteration in the blood crasis. Cases illustrative of this particular point, and most practical remarks upon its pathology, are given by MM. Huguier and Littré.|| Very interesting cases of abscess of the spleen, after fracture of the skull, leg, (compound) etc. are to be found in the *Lancet* for 1827–28, vol. ii., by Thos. Rose, Surgeon to St. George's Hospital.

There are only three conditions with which gangrene of the spleen can be confounded. These are:—

1. Apoplexy.
2. Abscess.
3. Post-mortem change of color.

That it was not apoplexy is clear from the description given by MM. Mignot and Lemaistre.¶ One of these cases was from continued fever, the other from a paroxysm of intermittent.

---

* Path. Générale, 1856, p. 545, (Chomel.)

† Epist. xxi. art. 29.

‡ Clinical Surgery, p. 154.

§ Practice of Physic, vol. i. p. 355.

|| Journal Hebdomadaire de Médecine, tome vii. p. 424, and tome iv. p. 449.

¶ Bull. de la Soc. Anat., 1848, (quoted by Valleix.)

"In each case there was an apoplectic spot, of greater or less extent, containing a half-liquid blood, and a kind of red, pulpy substance." This corresponds essentially with Choulant and Richter's account of the same lesion.*

It differed again from the appearances presented in abscess of the spleen, for "pyæmic abscesses (metastatic) form a single, circumscribed, peculiar deposit, which goes wedgelike from the circumference toward the centre; it is at first blackish-red, then yellowish, but with dark margins, and finally purulent."† Or, again, the spleen may be found "of a tawny color, soft, friable, presenting either traces of purulent infiltration, or scattered depots of pus, or a voluminous abscess."‡

It remains, in conclusion, to consider changes of color, particularly green coloration. "Green coloration of the tissues," observes Vogel,§ "is but rarely met with. It is sometimes observed in the lungs, the intestinal canal, and the muscles." Most of these tints depend upon changes in the body after death; some, he thinks, may be owing to the presence of sulphuret of iron, while others result from effects of putrefaction, unknown to us. He hazards the conjecture that many of these changes are owing to bile-pigment permeating the walls of the gall-bladder, and spreading, by imbibition, into the surrounding parts. "Further investigations must determine whether this kind of coloration of the dead body is seen in man at any distance from, or whether it is confined to, the immediate neighborhood of the gall-bladder."

In the case cited by Vogel, the discoloration was pretty general, and extended into the parts adjoining the gall-bladder. But in the case before the Society the change in color was small in extent, local, circumscribed, and at a comparative distance from the gall-bladder.

The intense general hue of inflammation of the organ, the line of demarcation, with its superincumbent vesicles, the peculiar fetid odor, and the character of the mass contained in the spleen, forbid us then to confound it with any of the above conditions.

*Cancer of the Great Omentum, Pyloric Extremity of the Stomach and the Small Intestines, with Fibrous Tumors of the Uterus.*—DR. DARRACH presented these specimens, which were removed by Dr. Barlow Darrach, at an autopsy upon a patient of Dr. Fricke, of this city. The patient was a female, of German extraction, married, and had had one child, a daughter. She was a tall, gaunt person, forty-eight years of age,

---

* Choulant and Richter, Path. und Ther., p. 970.

† Choulant, p. 970.

‡ Valleix, Guide du Médecin Practicien, tome iii. p. 275.

§ Path. Anat. Human Body; Phil. 1847, p. 360.

and of a nervous temperament. For the last fourteen years, at least, she has not been known to have had any *severe* sickness. She had always suffered from a dry, parchment-like state of the skin on the arms and lower limbs; and has also had frequently slight attacks of dyspepsia, accompanied with eructations of wind and loss of appetite, which always yielded readily to some mild remedies, such as an aperient pill, etc. She was a hard-working woman, having for many years been a cook in a large eating establishment.

The sickness, which terminated in her death, commenced in July last. She then complained of great pain in her back, and shortly afterwards she had numerous and large alvine discharges, accompanied by tenesmus. Her stools were changeable in appearance; sometimes mucoid, at other times presenting well-formed discharges. In the latter part of her affection, the intestines were constantly distended with gas, so much so as to prevent a satisfactory examination of the abdominal viscera. She still suffered much from eructations of wind, and a diffused pain over the abdomen.

The above symptoms gradually augmented throughout July and August, the pain becoming the prominent symptom. A very severe attack of palpitation of the heart occurred at the end of the month of August. Dr. Fricke thought this to be owing to the action of quinine, which she was then taking. This palpitation was so alarming that the woman was thought to be dying; but relief was obtained after the administration of valerian. Now followed great insomnolency and constant vomiting for two weeks, which was considered to be owing to some obstruction in the bowels. The tongue was natural; the patient had no cachectic appearance.

Autopsy.—The abdominal cavity alone was examined. The liver was healthy; the stomach was thickened at the pyloric end, which thickening, from microscopical examination, was proved to be cancerous; no cancerous elements could be discovered, however, in the other parts of the thickened walls of the organ; from which it was inferred that the thickening, except at the pyloric end, was owing to hypertrophy. The great omentum was also thickened and contracted from cancerous deposit, which was undergoing oily degeneration; the small intestines, for the distance of about one or two feet from the ileo-cæcal valve, were thickened and contracted by a deposit which had all the characters of cancer. Beneath their peritoneal investment were numerous small circumscribed deposits, resembling the deposits of tubercles, but which were found to consist of cancerous elements. The kidneys were healthy; the uterus had four fibrous tumors growing from its fundus, the largest one, being about the size of a large walnut, had undergone peripherally calcareous degeneration; the appendages of this organ were healthy.

*Cancer of the Liver and Supra-renal Capsules; no Bronzing of the Skin.*—DR. DA COSTA presented, for Dr. Wm. King, a cancerous liver of enormous size:—Frederick Harkin, aged thirty-five, a native of Ireland, and a farmer by profession, was admitted into the St. Joseph's Hospital on the 19th of August, 1858, suffering with an abdominal tumor, extending from the right to the left hypochondriac region, and downward some inches below the umbilicus; it was hard, knotty, and immovable. The tumor had been noticed by the patient, for the first time, about three months previous to his admission. Upon making inquiries as to its first appearance, he persisted in stating that it came on suddenly after drinking a quantity of water; while his wife, with about equal accuracy, attributed it to a stroke of lightning. The symptoms, during the whole course of the disease, were not well marked, and it was impossible, with any certainty, to form a diagnosis. At the Pennsylvania Hospital, where the patient had been under treatment for a month, no positive opinion as to the nature of the affection had been given. At the St. Joseph's Hospital, his attending physicians, Drs. Keating and Keller, came to no definite conclusion as to the true character of his disease.

Vomiting was one of the most prominent symptoms; it was generally induced by a full meal; the matter ejected consisted, first, of the food he had taken, and afterwards, of a glairy mucus. The liver appeared to perform its functions up to the time of his death; no symptoms of jaundice appeared; the bowels were regular, and the stools bilious; the patient felt no pain in any portion of the chest or abdomen; nor did pressure occasion any. Debility was one of the most distressing symptoms of which he complained; although not confined to his bed while under treatment, he expressed little desire to be moving about. Emaciation was present in a slight degree, but the peculiar cachectic condition of the system, common to cancer, was entirely absent; the color of the skin was natural. From the 19th of August up to the time of his death, which occurred on the morning of the 24th of September, he suffered only from debility, the weight of the tumor, and vomiting after meals. On being asked by the attending physician, a few days before his death, if he was improving, he expressed the opinion that the tumor was much lighter.

On the evening of the 23d he passed a good night. At about four o'clock on the following morning he arose from his bed, but became suddenly so weak that he had to be assisted back by the nurse. After having been placed in bed, he vomited, at first water, and afterwards mucus; the vomiting was succeeded in a short time by insensibility; the pulse was frequent, but small; the breathing labored, and the face of a livid hue. In this condition he remained until twelve o'clock, when he died.

Autopsy.—Six hours after death an examination was made, which revealed the following conditions of the viscera:—The liver formed in the abdomen a large tumor, weighing twelve pounds, which occupied the right hypochondrium, the epigastrium, and the left hypochondrium, and descended some inches below the umbilicus; its anterior and posterior surfaces were covered with a number of whitish indurated masses, evidently cancerous; these deposits were more numerous on the left lobe and on the edges; the liver measured thirteen inches in length, twelve inches in breadth, and four inches in thickness through the centre; the gall-bladder was distended with bile. The stomach was much displaced, but in a healthy condition; the pyloric extremity rested on the spleen, this organ being much increased in size, and of a dark color; the transverse colon was situated at the lower portion of the abdomen, as far down as the hypogastric region; very little omentum remained; the pressure of this immense liver no doubt had produced its absorption. On examining the mesentery, the glands were found much enlarged, and of a bright-red color; the remaining abdominal viscera were in a healthy condition; the epigastric and internal mammary veins were much enlarged, but there had not been the slightest effusion into the peritoneal cavity. On examining the thorax, the lungs were found to contain deposits, probably cancerous. The heart was natural; no lesion was found which could account for the sudden death. It is, indeed, a remarkable circumstance in this case, that, notwithstanding the cancerous disease of the liver, no pain was felt, nor were the functions of that organ interfered with. The supra-renal capsules were both diseased, and infiltrated with cancer.

DR. KEATING, who had attended the patient for some time, stated that the extent of the swelling in the abdomen had almost precluded the idea of an affection of the liver. The appetite of the man was enormous; the stools were solid, and of natural color and appearance. There was not the slightest trace of bronzing of the skin; even the peculiar hue of the countenance, met with in cancer, was absent. The supra-renal capsules were microscopically examined, and presented undoubted evidences of cancer. The patient had employed iodine friction over the abdomen, which he thought had reduced the size of the organ.

DR. MITCHELL spoke of the frequent absence of the peculiar color in cases of cancer, and suggested that there might be, as it were, two sets of cases, one with and one without this symptom.

DR. ADDINELL HEWSON was now attending a patient in whom he had diagnosed cancer of the liver, and whose complexion did not in any way indicate cancer.

WEDNESDAY EVENING, October 27th, 1858.

The President, DR. LA ROCHE, in the Chair.

*Cancer of the Liver.*—DR. ADDINELL HEWSON exhibited specimens removed from the body of Mrs. C., to whose case he had alluded at the previous meeting. He had visited Mrs. C. for the first time on the 1st of October, and found her in bed, exceedingly pale, weak, and emaciated, and was informed by her daughter that she had been suffering from diarrhœa for about four months, and that very recently the alvine evacuations had become bloody, and caused them alarm. She had rejected all nourishment from her stomach during the whole of her illness. Her tongue was very smooth and pale; the lungs and heart, on examination, were found normal; the pulse feeble and frequent, 140. On passing the hand beneath the bedclothing and over the abdomen, he was surprised to find this region tumid and bossilated, as though the intestines were distended by hard, scibulous masses of fæces; this hard, bossilated condition extended from beneath the ribs all across the abdomen, and below the umbilicus into the lower regions of the abdomen; the edge of this hardness was very nodular, and, by pressing on the attenuated integuments of the abdomen, the fingers could be passed under this border; the lower part of the abdomen was dull; this was especially marked in the right illiac fossa. The patient complained of no pain in the region of the tumor; she suffered occasional colicky pains when at stool; the alvine evacuations were about four per diem, thin, but not deficient in bile; the tinging with blood was found to proceed from hemorrhoids. The patient had never observed the tumor of the abdomen before the date of her present illness; she was fifty-eight years of age; had always been thin and pale; her parents lived to an advanced age; she did not know of either of them having died of cancer; she had lost two sisters with phthisis; she was a widow; had had two children, the younger about twelve years old. Her health had been failing ever since her change of life, (at fifty-two years of age,) at which time she lost her husband; since then she had been much reduced in circumstances; she had never had any uterine discharge, or trouble with her kidneys. She rallied somewhat under the use of stimulants, but died fifteen days after she was first seen by Dr. Hewson.

Autopsy twenty-two hours after death.—Rrigor mortis well-marked; slight straw tint of surface; emaciation extreme. Abdomen—enormously distended; a hard, knobby tumor could be readily felt through the walls occupying the right hypochondriac and lumbar, the epigastric, and part of the umbilical, and the left hypochondriac regions; on making the section of the walls much gas escaped, and about three quarts of fluid were found in the cavity of the peritoneum; the tumor was found to be of the liver, which was bound by old adhesions to the walls of the abdomen on the right; the organ itself was greatly enlarged, extending up under the ribs as high as the fourth rib; its surface was studded with cancerous masses, and similar masses were distributed through its structure; the portions unoccupied by them were in a state of fatty degeneration; the organ weighed, when removed, nine and a half pounds; the gall-bladder was much distended with bile; the stomach had been pushed over by the liver into the left side, and made to occupy an almost vertical position in the left hypochondriac and lumbar regions; it was healthy as to structure. Under the liver, in the right side, was found the colon, doubled on itself at the angle of the ascending and transverse portions, bound down by adhesions, so as to nearly obliterate the canal, and in the centre of these adhesions was found a depot of about a drachm of thick, greenish pus, with the walls of the gut so much attenuated that they gave way at one point; the alimentary canal, beyond this, was healthy; in the vicinity of this the peritoneum was studded with hard masses of the size of a pea; the same were found in the right broad ligament, which was bound to the cæcum; and under these, in the iliac fossa, another collection of pus, of about a drachm in quantity; uterus and ovaries were free from disease, and both kidneys were in a healthy state, although unusually pale. Thorax—there was about an ounce of fluid in the pericardium; the heart was healthy; the lungs appeared natural; they were œdematous and frothy, and at the apex of the right lung there was a little hard mass of the size of a shot; nothing could be found in this to indicate cancer; the masses in the liver were observed, on a microscopic examination, to be cancerous.

*Ossification of the Splenic Artery.*—The preparation exhibited to the Society by DR. HALL was removed from a man, aged seventy-eight, who died of phthisis, in the Pennsylvania Hospital. He had been a sailor all his life, of drinking habits, and was utterly broken down in constitution. He was over a year in the House, and at the time of his death was little more than skin and bone.

Prior to his death it was evident that the right brachial, with its branches, were ossified—this could be felt at once on passing the fingers over the course of the branches.

At a post-mortem inspection, the aorta, at and just below the curve, was found studded thickly with osseous scales. The brachial artery of the right side also contained calcareous patches, while the radial and ulnar arteries were in parts quite rigid with the new formation. (These preparations were shown to the Society.)

The splenic artery was ossified throughout its course; a lesion which is rare. Dr. Bristowe,* of St. Thomas's Hospital, reports a case of "A female, aged sixty-seven, dead from dry gangrene of the foot. The arteries of the thigh and leg were ossified. The splenic artery was ossified throughout. The spleen small and natural."

Morgagni, also, quotes the case of an old man, in whom "the aorta at its bifurcation was ossified, and the splenic artery, from its commencement to its entrance into the spleen, was considerably dilated, and consisted almost wholly of bone."

DR. PACKARD alluded to the fact that the term ossification was very vaguely used; true bone being among the rarest of pathological formations.

DR. GROSS did not agree with Dr. Hall that ossification of the splenic artery was a very unusual occurrence. He had met with it not unfrequently, more often in the male than in the female. In the male, indeed, all arteries are more frequently ossified, a fact which he had had opportunity of observing himself, and which, on inquiry, he had found borne out by several pathological anatomists in this country with whom he had corresponded on this subject. Dr. Gross then exhibited a specimen, of a stellate appearance, which he believed to be true ossification of the valves of the aorta of an ox.

*Examination of Black Vomit.*—DR. PACKARD read the following:—

During the past summer I have had two opportunities of making microscopical examinations of specimens of black vomit.

One of these was given to me by Dr. La Roche, immediately after its ejection. Examined under a magnifying power of 360 diameters, were seen—

1. Epithelial cells of the squamous variety.
2. Bodies resembling epithelial cells in various states of distension, some apparently filled with coloring matter of different shades, from light yellow to black. A few of these were perfectly opake; many of the rest were highly refractive.
3. Some brownish-yellow, nucleated cells, about twice the size of lymph-corpuscles.
4. Some very small reddish cells which may, perhaps, have been altered blood-corpuscles.
5. Many fine granules.
6. A few quite minute oil globules.

In one of the portions examined there were several masses of what seemed to be partly digested food.

---

* A Treatise on the Structure and Use of the Spleen. By Edward Crisp, M.D.; London, 1854, p. 150.

The other specimen was taken from the stomach of a sailor who died, with all the signs of yellow fever, in the Pennsylvania Hospital. The power employed was 472 diameters.

1. Epithelial cells in vast numbers, arranged in masses. Very few of them were squamous; the immense majority were columnar, and ranged side by side.

2. A great many fat globules, of all sizes.

3. Free nuclei.

The liver of this patient was very decidedly fatty, both in its grosser characters and under the microscope.

The same degeneration was very marked in the liver of another patient in whose case a similar diagnosis had been made.

DR. S. HENRY DICKSON inquired whether any of the members had met with the ruptured capillaries which are described by Blair as present in black vomit. In examination of black vomit made at Charleston none were detected.

DR. LA ROCHE, and several other members of the Society, stated that they had never met with them.

*Fatty Degeneration of a Nerve, in a Case of Tetanus.*—DR. PACKARD continued: I have also made an examination which may have some interest, as perhaps bearing upon the pathology of that very formidable disease, tetanus; not explaining it, but possibly giving a ray of light in regard to it in this particular case.

The patient was a man in whom, after an amputation of the forearm, sloughing of the stump had occurred, and subsequently tetanus. By the sloughing was laid bare a portion of the ulnar nerve, which was excised by Dr. Pancoast with a faint hope of arresting the symptoms. A portion of the excised bit of nerve seemed to the naked eye to be healthy, the rest was abnormally vascular.

The former, under the microscope, was proved to be actually in a normal state; the latter was throughout in a condition of well-marked fatty degeneration. Repeated examinations of various specimens placed the fact of this difference of state beyond a doubt.

It would be absurd to infer from this single fact that tetanus is always connected with local inflammation somewhere in the nervous system, for the degeneration was here probably an inflammatory change; but the only way to arrive at the pathology of so rare a disease is to carefully preserve every item of information, and it is only as such an item that I present this isolated observation.

DR. HEWSON stated some facts in relation to this case of tetanus, which he thought important in connection with Dr. Packard's remarks. The amputation had been performed by himself on the case, in the Pennsylvania Hospital, for a severe gunshot wound, which had caused extensive comminution of the bones of the forearm, and burning of the integuments of the arm. The amputation was of the circular form, and was performed about two and one-half inches below the elbow, the patient being placed under the influence of ether. Excessive inflammatory reaction ensued in the burnt surface, and a gangrenous slough followed, which not only involved the cuff of the stump, but the integuments of the arm considerably beyond the limits of the original burn. It was on the separation of this slough that the tetanus set in.

The portion of nerve removed by Dr. Pancoast lay partly in a pouch formed by an accumulation of pus beneath the integument, between the internal condyle and olecranon, and partly in sound tissue above. The latter was what Dr. Packard has mentioned as normal, while the former was what he has described as in a state of fatty degeneration. The patient lived some time (about ten days) after the operation, but did not seem to have been benefited by it.

*Ulceration of the Larynx and Trachea; Death from Œdema of the Glottis.*—DR. HALL presented, for Dr. Albert H. Smith, a larynx and trachea removed from Bridget Nolan, aged fifty, native of Ireland, a housekeeper, who entered the Pennsylvania Hospital October 18th, 1858. She stated at the time that she had had a cough for two years, having been attacked first with croup, of which she had had several attacks since. When admitted she was apparently in fine health, stout and ruddy; the only evidence of disease being an occasional stridulous inspiration. On examination of the chest, slight sonorous and mucous rales, with a whistling sound in the trachea, were found. After admission, she was observed to have a stridulous cough, coming on in paroxysms, and lasting several hours at a time, with violent dyspnœa. These symptoms continued without change until about two weeks ago, when she was attacked suddenly at midnight with suffocation, and, before she could be reached, was dead.

Autopsy.—The larynx was found completely closed, with an œdematous swelling of the glottis, both above and below which the course of the larynx and trachea was blocked up with tenacious mucus. From the glottis to the bifurcation of the trachea, principally upon the posterior wall, were numerous large, deep ulcerations, with indurated edges.

There was no reason to suspect any syphilitic taint, there being no evidence of it in any part of the body; the vagina, uterus, ovaries, and other organs, were carefully examined, and found healthy. She was a married woman, with a very respectable family of children.

DR. GROSS nevertheless inclined to the belief that the ulcerations were of a syphilitic nature, partly because there was no evidence of tubercle, which might account for the ulcerations, and partly because they were multiple and presented raised and indurated edges.

*Rupture of the Spleen; Collapse of the Lung.*—The Secretary presented, for Dr. J. C. HARLAN, a ruptured spleen, removed from William Gordan, aged eleven, who was admitted into the St. Joseph's Hospital early on the morning of October 15th, suffering from injuries inflicted by a recent accident. While playing in the street he was knocked down and ran over by a butcher's wagon, one wheel of which, he stated, passed over his abdomen and another over his chest. He complained of some pain in the lower part of the abdomen, just above the pubes, but suffered mostly from pain in the chest and difficulty of respiration. His stomach was very irritable, and could not be made to retain anything without great difficulty. A great swelling of the face and neck was obvious at the first glance, and on further examination was found to extend over the chest and abdomen, and to be evidently caused by an enormous effusion of air. No other injury could be discovered, and he expressed himself free from pain in the other parts of the body.

It was supposed that a rib had been fractured, and that the emphysema was due to a wound of the lung by a sharp point of the broken bone. The chest was not minutely examined, as the great pain and prostration forbade it, and indeed the emphysema was so extensive as to render anything like an accurate and satisfactory examination impossible.

Stimulants and opiates were administered, a sinapism applied over the epigastrium, and a bandage placed around the chest. The bandage gave great relief at first, but was afterwards removed, as the boy thought it increased the oppression. He reacted pretty well, and in the afternoon breathed rather more freely and suffered less. He slept at intervals during the early part of the night, but about one o'clock he was seized with a pain across the upper part of the abdomen, so violent as to cause him to scream aloud. It was relieved by large doses of opium and a warm mush poultice applied over the part, and he slept pretty well until morning. During the night a scrotal hernia was observed, which, it may be confidently stated, did not exist at the time of his admission, as the pain above the pubes led to a careful examination. It was probably a result of the continued pressure of the air effused into the abdominal parietes. It was reducible, but returned immediately when unsupported. The next day the pain in the abdomen continued, and was rendered supportable only by the administration of opium, and the constant application of warmth and moisture by the poultice. Toward evening he became delirious, and died between two and three in the morning of the third day after the accident.

Post-mortem examination.—The left lung was in a perfectly healthy condition. The right was completely collapsed and congested, but not wounded, and there was no fracture of the ribs, sternum, or clavicle, nor injury of the bronchi or trachea. The apex of the lung had been confined by an old pleuritic adhesion, which it seemed as if the sudden and violent pressure upon the chest had broken loose. The pleura itself was torn. In the abdomen there were no signs of general peritonitis. The small intestines, liver, and right kidney, were perfectly healthy. There was an effusion of blood in the left hypochondrium. The spleen was ruptured, and the left kidney, transverse colon, and pyloric extremity of the stomach were in a condition bordering on gangrene. As he passed clear urine, it must have been secreted entirely by the right kidney. The liver, though not injured, is worthy of inspection. The curious fissures sometimes observed in it exist here in unusual number. There are also a number of marks that have the appearance of cicatrices, and in one instance a small fissure may be seen at the end of a cicatrix, as though the remains of a large one partially closed.

A discussion arose as to the mode of accounting for the emphysema and the collapse of the lung, since no external injury of the chest had been found. On motion, the case was referred to Dr. Wm. Keller, to obtain additional particulars.

*Case of Gangrene, Probably from Arterial Obstruction.*—The following case, recorded by DR. HENRY HARTSHORNE, was presented through the Secretary: In October, 1857, I was called to see, with Drs. Halsey and Hazzard, a man of about forty-five years of age, of previously good health except asthmatic attacks, suffering, at the time of our visit, with gangrene of the left foot.

The history of the case was, briefly, as follows: While walking in the street, he was suddenly attacked with intense pain at the upper part of the thigh, somewhat deeply seated. This continued for many hours, obliging him to retire to bed. Within twenty-four hours from the time of the commencement of the pain, a marked coldness was perceptible in the limb affected. This increased gradually, with equally gradual diminution of sensibility in the foot, and, in three or four days, change of color, commencing in the toes and spreading slowly up to the ankle. No evidence whatever of erysipelatous or other form of inflammation in the foot existed. The temperature, sensibility, and appearance of the other limb were entirely natural. I saw him on the fourteenth day of the illness, and his death took place on the seventeenth.

The symptoms, at the time of our visit, were those of constitutional irritation, and moderate but increasing prostration, with blackness of the whole foot and part of the ankle, and total insensibility of the foot. The most striking feature of the case, however, was the alteration in the arterial circulation of the limb affected. As was repeatedly ascertained upon careful examination by Drs. Halsey, Hazzard, and myself, the pulsation of the popliteal and femoral artery (that of the tibial arteries being imperceptible) was not only, as might be anticipated, feebler, but very decidedly and constantly *slower* than that of the corresponding vessel in the other limb. Its rate was, in fact, about *half* that of the other.

The death of the patient took place rather suddenly, with symptoms parallel to those of the attacks to which he had been subject, and which were described as asthmatic. No autopsy was allowed.

Having had the opportunity to make but one visit to him, my attention was not, at the time, called to the condition of his heart, otherwise some ground might be afforded for accepting or rejecting the only hypothesis which suggests itself to me to account for the occurrence and course of the case. This hypothesis is, that the so-called asthmatic symptoms were those of dyspnœa from cardiac disease; that, possibly, during a subacute exacerbation of endocarditis, a fibrinous mass had escaped into the aorta, and travelled as far as the femoral artery, upon which it acted as a plug. The irritation produced by its presence might account for the pain at the commencement of the attack. The suddenness of the patient's death, moreover, at an earlier period than was to be expected from the depressing influence of the gangrene alone, might, perhaps, have resulted from the consummation of the cardiac disease itself. The age, constitution, and habits of the patient, at all events, along with the train of symptoms, removed the case entirely from the category of senile affections, and as positively did the local conditions prevent the diagnosis of erysipelas gangrenodes. If, therefore, the hypothesis above stated be rejected, what theory of causation can be substituted for it?

---

WEDNESDAY EVENING, November 10th, 1858.

The President, DR. LA ROCHE, in the Chair.

*Idiopathic Gangrene of the Groin.*—A case presented by DR. ADDINELL HEWSON.—Dr. H. was called to see the infant of Mrs. D., residing in Hide's Court, on the 21st of October. He found the child a pale, feebly-nourished little thing of nine months, suffering from teething and diarrhœa; the latter had been annoying her, off and on, through the whole of the past summer, when she cut the two lower incisors; the upper incisors were still very deep, but the gums much swollen. She had been under the care of a practicing apothecary during the whole of her sickness. A

few days before he saw her for the first time, she had been much disturbed by nausea and vomiting, and by an increase of the diarrhœa; the discharges on the napkin were very white and thin, quite watery; the abdomen, when she was first examined, was flaccid, soft, and nowhere tender on pressure; she voided her urine freely. The diarrhœa and sickness yielded readily to treatment; the former had nearly ceased at the end of the third day from the Doctor's first visiting her, when the mother noticed, in putting her to bed in the evening, that she was very fretful, flinching and crying, as from acute pain, when the right hip-joint was moved. On examining the groin of this side, she also observed a livid tumefaction, which she was confident was not there in the morning when she dressed her; this lividity extended from the anterior superior spine of the ilium along the groin to the vulva. On the following day it had much increased, and the child seemed in great pain, feverish, but much exhausted; there was hardness and heat of surface extending up to the umbilicus, and the centre of the morbid action was dark-brown, this color extending the whole length of the groin, and for a width of half an inch; there was no fluctuation, and the sensibility of the brown surface seemed to be diminished. The color here became rapidly darker, and in the course of four days the part was black, soft, and had begun to be separated from the surrounding structures, a considerable quantity of grumous blood escaping from beneath the slough. The child sank under the process of separation of the slough, and died on the 2d of November, so quietly in her mother's arms, that she was not aware of her dissolution at the time.

*Autopsy*, made thirty-two hours after death.—The destruction of tissue had extended through the subcutaneous cellular tissue, and the contrast of the marble-whiteness of the child's body with the black sphacelated parts was very striking; there was no incarcerated hernia or other local source found that could account for the disease. A dissection of the vessels in the groin was not permitted, an examination of the viscera of the abdomen being all that could be obtained; here everything was found in a healthy state.

DR. WOODWARD said that he remembered a case similar, in some respects, to that of Dr. Hewson. It occurred in Blockley Hospital when he was an Assistant-Resident in that institution. Measles had been endemic in the Children's Asylum, and many of the little patients subsequently suffered from severe bronchitis or pneumonia; after which a number of cases of *gangrena oris* made their appearance, all of which, so far as he recollected, terminated fatally. While this state of things existed, his attention was called by the nurse to a small, gray, depressed, gangrenous spot in the groin of a little girl, about six or seven years of age, who had apparently recovered completely from her attack of measles. In spite of treatment, this spread rapidly, and she died in forty-eight hours. At the time of her death an extensive moist slough occupied the whole of that groin as well as the mons-veneris and orifice of the vagina. The symptoms prior to her decease were those of extreme depression. A post-mortem examination was made, but, so far as he recollected, there was no marked lesion in any of the abdominal or thoracic viscera.

*Constriction of the Pylorus from Hypertrophy of its Fibrous and Muscular Coats.*—DR. DA COSTA presented, for Dr. Wm. Moss, a specimen of narrowing of the pylorus, with Dr. Moss's account of the case:—

Mrs. M., aged forty, came under my care on the 20th of last September, at which time she had been suffering about five weeks from the following symptoms:—Pain at the pit of the stomach, not increased by pressure; a "dead," heavy sensation throughout the abdomen, and vomiting shortly after meals, the matter vomited consisting of the ingesta only, containing neither bile, glairy fluid, nor blood. For some months she had been subject to attacks of indigestion, and had been steadily losing flesh and strength; she complained especially of great weakness in her legs; her eyes were sunken and unnaturally brilliant, and the expression of her countenance was utterly despondent; she had a slight cough; her bowels were obstinately constipated; the tongue smooth and red; pulse 100, feeble and irregular. On physical examination, I found slight dullness at the apices of the lungs; heart's size and action natural; liver not enlarged; no signs of aortal aneurism, or of tumor in the abdomen, except the slightest possible resistance to the finger over the pylorus, unaccompanied by dullness on percussion; urine of healthy specific gravity and constituents; no pain on pressure over the spinal column.

The diagnosis arrived at by Dr. Da Costa and myself, more by exclusion than from any distinctive symptoms which the case presented, was, that there existed an obstruction at the pyloric orifice, not caused by cancer nor the result of ulcer at the pylorus. That the lesion was not an ulcer of the stomach, was clearly shown by the dull, heavy character of the pain, its diffusion over the abdomen, its not being increased by pressure, and the absence of blood in the matter vomited; while the non-existence of the symptoms of ulcer in the previous history of the case tended to prove that it was not likely that a cicatrix from a former ulcer could be the cause of the malady.

We inferred that cancer was not present, because, in the state of exhaustion to which the patient was reduced, we would have had, in addition to the actual symptoms, vomiting of glairy mucus and of the characteristic coffee-ground fluid of cancer, and, in all probability, the presence of a tumor perceptible to the touch.

As to what was the real cause of the obstruction, in the absence of all positive symptoms, we could only hazard a supposition. It might have been owing to a tumor pressing against the pylorus from behind; and since the pancreas was the only one of the neighboring organs of whose normal size we were not certain, it seemed not improbable, that the enlargement might be of that organ. Yet this was a mere supposition. It would have, to some extent, accounted for the numbness of the thighs, by pressure on the nerves; but the absence of any signs of a tumor invalidated it.

The history of the case may be recounted in very few words. The vomiting after meals was an almost incessant symptom, ceasing once only, on the 23d of September, without apparent cause, since it recommenced a couple of days afterwards under the use of the agent which I flattered myself was controlling it, hydrocyanic acid, to end only with the life of the sufferer. I was equally unsuccessful in combating it with creosote, bismuth, and the opiates.

On the 29th she was seized with severe lancinating pains under both patellæ, unaffected by pressure or motion, and accompanied by pricking sensations and numbness in the legs, with great difficulty in moving them. The pains gradually ceased, together with the pricking, but the loss of motion and numbness increased daily.

October 3d.—Tongue coated; increased pain in epigastrium, and acid eructations, which yielded to the employment of bismuth and sod. bicarb. During all this time the constipation was most obstinate, requiring the daily use of laxative enemata. Her extremities were cold; her pulse was scarcely perceptible, ranging from 110 to 130 beats in the minute, and she could obtain with difficulty a few moments of sleep.

On the 17th I first noticed that her mind occasionally wandered, so slightly and rarely, that perhaps this state may have existed for several days before it attracted my attention. The constantly increasing numbness and weakness of her lower extremities terminated in complete paralysis about a week before her death; the abdominal pains and vomiting increased in severity; she complained of headache and occasional febrile attacks, and during the last few days of her life her pulse became so faint and frequent that I could not count its beats. She retained her mental powers perfectly, with the exception of the occasional above-mentioned aberrations, until the time of her death, which took place on the 26th of October, about ten weeks after I first saw her.

Examination twenty-four hours after death.—Thorax, heart, and pericardium healthy; old adhesions of pleura over tuberculous deposits in apices of both lungs; small cavity in apex of right lung; the pleura and pericardium contained no fluid. Abdomen—adipose tissue of walls about three-quarters of an inch thick; the liver, spleen, pancreas, and urino-genital organs healthy, as were the choledoch and pancreatic ducts; gall-bladder empty; the stomach was collapsed, very slightly, if at all, enlarged, empty, and not thickened; its mucous coat was smooth, clean, not thickened, and presented no signs of congestion or of inflammation; the pyloric orifice was so narrowed that the finger could with difficulty be forced into it, and its mucous lining lay in folds, like the mouth of a bag; there were no traces of ulcers or cicatrices, and the narrowing proved to be due to a thickening of the submucous and muscular coats. The rest of the intestinal canal was healthy; there was about a teaspoonful of jellylike substance lying loose in the stomach near the pylorus, which, under the microscope, consisted of granular matter and a few epithelial cells; it was, I imagine, the thick beef-tea of which she had partaken shortly before her death. Nothing was found to explain the paralysis of the lower extremities, and I was not permitted to examine the brain or spinal cord, to some lesion of the latter of which it must doubtlessly be attributed. On dissection of the pylorus its mucous coat was unaltered, and easily detached from the subjacent coat; the fibrous and muscular coats were uniformly thickened, with none of the characteristics of scirrhus to the eye or touch; examined microscopically, the thickened substance was found to be composed of fibro-areolar tissue, containing formative cells and muscular fibres of organic life.

The absence of cancer-cells under the microscope, the uniformity of the enlargement, its softness to the touch, and the presence of tubercle in the lung, were sufficient evidences that the thickening was due, not to a cancerous deposit, but to hypertrophy of the normal constituents of the part.

The points which occur to me as most worthy of notice in this case, are, the impossibility of diagnosing with certainty a stenosis of the pylorus dependent on hypertrophy of its submucous and muscular coats, and the exceeding rareness of this disease, or rather the rare mention which has hitherto been made of it.

The physical and rational signs of constriction of the pylorus, whether from mere hypertrophy of its coats or from a cancerous thickening in its first stage, that is, before ulceration or erosion of the mucous coat has taken place, and before the tumor has attained sufficient size to be perceptible to the touch, are identical; so that to the difficulty, so dwelt on by all writers on the subject, of diagnosing cancer of the pylorus in its earlier stages, we have superadded the utter impossibility of distinguishing between it and the lesion now under consideration.

As to the infrequency of this lesion, I can find but few writers, even among those who have especially studied the diseases of the alimentary canal, who seem to have suspected its existence. Budd, Chambers, Valleix, Grisolle, and Andral make no mention of it. Rokitansky (*Pathological Anatomy*) simply alludes to hypertrophy of the gastric membranes as one of the causes of stenosis. Habershon describes, what seems to me to be the same affection, a fibroid degeneration of the pylorus, a fibrous deposit commencing in the submucous cellular tissue, obstructing the valve and followed by muscular hypertrophy, with symptoms identical with those of cancerous obstruction.

Of the later writers, Bamberger* enumerates it among the causes of stenosis of the pylorus, carefully pointing out its exceeding infrequency. Hennoch† mentions it in exactly similar terms, and refers to Brandt on Stenosis of the Pylorus, and Dittrich, the latter of whom had met with it in adults and in children under one and a half years of age.

Cruveilhier, (*Anatomie Descriptive,* article ESTOMAC,) so distinctly asserts the existence of this lesion, that I may be excused for translating, in conclusion, the entire paragraph:—

"In the examination of several patients, who, toward the close of their life, had presented all the symptoms of stenosis of the pylorus, I found no other alteration than a very remarkable hypertrophy of the pyloric ring. I cannot decide whether this stenosis were cause or effect of the continual vomiting experienced by the patient; and I scarcely dare assert that these hypertrophies of the pyloric ring have been frequently shown to me as scirrhous stenoses, by persons but little versed in the knowledge of healthy and morbid anatomy."

---

WEDNESDAY EVENING, November 24th, 1858.

Vice-President, DR. STILLÉ, in the Chair.

*Cancer of the Pylorus.*—DR. HALL exhibited a specimen of cancer of the pylorus, removed from a patient of Dr. De Benneville of this city. For many years the patient had complained of a soreness and disagreeable dragging sensation in the region of the stomach. For a month or more before his death there was but little acute pain in the stomach, but rather a feeling of soreness after the eructation of food, which followed within an hour of its ingestion.

Post-mortem examination fourteen hours after death.—Rigor mortis not well marked; body extremely emaciated; right arm amputated at shoulder-joint, a good stump, with a firm and not extensive cicatrix. A large, hard tumor in the epigastric region, to be felt readily on palpation through the

---

* Uber die Krankheiten des Digestions Apparatus; Virchow's Handbuch.

† Klinik der Unterleibs Krankheiten.

attenuated abdominal parietes. Head not examined. Lungs and heart perfectly healthy; liver also normal. Spleen—capsule thickened, and opake patches from old inflammation. Kidneys healthy. Stomach—normal as to position in cavity of abdomen and adjoining organs; nodulated masses upon the superior curvature of the organ; contents not unusual; color of membrane natural, save in the pyloric extremity, where there were streaks of bright injection; no mamillation of the membrane; no softening, as evidenced by tearing up of strips; thickness at pyloric extremity seven-tenths of an inch, at cardiac extremity one-fifth of an inch; cardiac opening not constricted. Pylorus open, the index finger could pass freely into it; the mucous membrane puckered at the orifice. Pyloric orifice three-tenths of an inch in diameter. Commencing at the pylorus was a scirrhous mass extending three and a half inches in the long axis of the stomach, and four and seven-tenths inches in the transverse or short diameter of the organ. At its highest point it had an elevation of three and one-tenth inches above the mucous membrane.

Under the microscope were found well-marked cancerous elements. The surface of the tumor had undergone serpiginous ulceration in spots; its general hue was light yellow, with here and there a yellowish-green shading. The pancreas was healthy.

DR. WOODWARD made a few remarks in connection with the *homologies of cancer*, which he regarded as a malformed new formation of connective tissue, as suggested by Wedl. One of the difficulties to the reception of this doctrine was the supposed absence of forms in embryonic development, approximating to those of cancer. It was stated, however, that in the young connective tissue of fœtal marrow, cells were often observed approximating to the forms usually regarded as diagnostic of cancer. Diversity in shape, large size of nucleus, and vesicular nucleolus being clearly observable; cells with double and even plural nuclei, besides the characteristic many-nucleated marrow cell, are to be seen.

A specimen exhibiting these appearances was shown, from the marrow of the tibia of a fœtus of five and a half months.

Certain conditions present in fœtal marrow will, perhaps, account for the shape assumed by these young connective tissue elements. These conditions are evidently—1. The large quantity of blastema afforded. 2. The modification of shape by mutual pressure. If to these be added a consideration of the stage of the development of the tissue of the embryo, which is that with which cancer is thought homologous, the significance of such forms as these will be at once apparent.

*Communication of the Appendix Vermiformis with the Ileum through a mesenteric gland; iron wires in the Omentum.*—DR. PACK-

ARD, in presenting these specimens, made the subjoined remarks: William Millbank, aged twenty-seven, a colored seaman, died in the Pennsylvania Hospital, June 26th, 1856, after an illness of about five months. When admitted into the institution, about two months previous to his death, he said that the doctors had told him that his disease had begun with typhoid fever; but his case was then evidently one of phthisis. The notes of its course are omitted as irrelevant.

Autopsy, fifteen hours after death.—Body excessively emaciated. Rigor mortis not very well pronounced. Brain softened; much serous effusion. Some tuberculous deposits existed around the optic chiasm, obliterating the fissure of Sylvius on each side. Lungs much congested throughout, exuding bloody or frothy serum on being cut into or torn; both apices healthy. The left lung was the one most diseased; the lower half of its bulk was converted into a yellow tuberculous mass, presenting several small anfractuous cavities, chiefly at its posterior part; just above this mass was some miliary deposit, of no great extent. In the right lung the middle lobe, and the lower portion of the upper, presented similar yellow masses; the lower lobe contained a great deal of miliary tubercle. Heart, liver, spleen, and kidneys, healthy. Intestines—in the ileum three of the agminated glands, of small size, were found ulcerated, and many others had an appearance as of cicatrization. The mesenteric glands were enlarged, especially about the ileo-colic junction; one of them had suppurated, and communicated with the interior of the caput coli, an ulcer existing in the mucous membrane; there were several other ulcers around the spot. The appendix vermiformis was adherent to the mesentery from inflammation; a perforating ulcer existed at its extremity; an ulcer in the ileum, about one inch above the ileo-colic junction, had also perforated the intestine, and both these ulcers penetrated into the cavity of a large, suppurated mesenteric gland. Whether or not this cavity had communicated with that of the abdomen, could not be determined. In the tissue of the great omentum, between its layers, were found two bits of rusty and crooked iron wire, two and one-half and one and one-half inches long. How these foreign bodies were introduced I cannot pretend to say, unless the patient had swallowed them at some period of his life.

I would call attention to the seat of the tuberculous deposit in the lungs in this case, and would inquire whether the fact of enteric fever, with its accompanying bronchitis, having been the exciting cause, may not have had some influence in this respect? Is there any tendency observable to the formation of tubercle, after enteric fever, about the inflamed tubes, rather than at the apex of the lung?

Another point of interest about this case is the discharge of the pus from an inflamed mesenteric gland into the caput coli. Did the ulceration of the mucous membrane occur independently, or did the gland burst at that point as a bubo would perforate the skin?

The communication of the appendix vermiformis with the ileum through the cavity of a suppurated mesenteric gland, although very easy of comprehension, is not of frequent occurrence, so far as I can ascertain. Whether this had anything at all to do with the passage of the wires into the abdominal cavity or not, is to my mind very doubtful.

Dr. Keller reported that he had made inquiry about the case of rupture of the spleen, and collapse of the lung, read at a previous meeting of the Society, and that he had been positively assured that the most careful examination failed to detect a fracture of the ribs.

Dr. Stillé suggested, that the laceration of the internal parts, unaccompanied by fracture of the ribs, might be explained by the elasticity of the thoracic walls, which in a child would readily yield, and this when the same force was sufficient to lacerate the resisting textures underneath.

Dr. Mitchell spoke of the evident effect which the abdominal walls and the intestines must exert in preventing ruptures of the internal organs during injuries received. They act, as it were, as air-cushions. Dr. Mitchell then related the particulars of a case of rupture of the liver. The patient, a bricklayer, fell from a considerable height and alighted on his feet. The shock was such that it tore the liver from its attachment, which was the more easily possible because the abdominal walls had been thinned, by a coarse truss, which was worn for the relief of a hernia. Thus the proper support of the abdominal viscera was wanting.

